# Wild and Cultivated Species of Rice Have Distinctive Proteomic Responses to Drought

**DOI:** 10.3390/ijms21175980

**Published:** 2020-08-19

**Authors:** Sara Hamzelou, Karthik Shantharam Kamath, Farhad Masoomi-Aladizgeh, Matthew M. Johnsen, Brian J. Atwell, Paul A. Haynes

**Affiliations:** 1 Department of Molecular Sciences, Macquarie University, North Ryde, NSW 2109, Australia; sara.hamzelou@hdr.mq.edu.au (S.H.); karthik.kamath@mq.edu.au (K.S.K.); matthew.johnsen@students.mq.edu.au (M.M.J.); 2 Australian Proteome Analysis Facility, Macquarie University, North Ryde, NSW 2109, Australia; 3 Department of Biological Sciences, Macquarie University, North Ryde, NSW 2109, Australia; farhad.masoomi-aladizgeh@hdr.mq.edu.au (F.M.-A.); brian.atwell@mq.edu.au (B.J.A.)

**Keywords:** drought stress, label-free quantitative shotgun proteomics, mass spectrometry, *Oryza australiensis*, *Oryza glaberrima*, *Oryza sativa*, parallel reaction monitoring

## Abstract

Drought often compromises yield in non-irrigated crops such as rainfed rice, imperiling the communities that depend upon it as a primary food source. In this study, two cultivated species (*Oryza sativa* cv. Nipponbare and *Oryza glaberrima* cv. CG14) and an endemic, perennial Australian wild species (*Oryza australiensis*) were grown in soil at 40% field capacity for 7 d (drought). The hypothesis was that the natural tolerance of *O. australiensis* to erratic water supply would be reflected in a unique proteomic profile. Leaves from droughted plants and well-watered controls were harvested for label-free quantitative shotgun proteomics. Physiological and gene ontology analysis confirmed that *O. australiensis* responded uniquely to drought, with superior leaf water status and enhanced levels of photosynthetic proteins. Distinctive patterns of protein accumulation in drought were observed across the *O. australiensis* proteome. Photosynthetic and stress-response proteins were more abundant in drought-affected *O. glaberrima* than *O. sativa*, and were further enriched in *O. australiensis*. In contrast, the level of accumulation of photosynthetic proteins decreased when *O. sativa* underwent drought, while a narrower range of stress-responsive proteins showed increased levels of accumulation. Distinctive proteomic profiles and the accumulated levels of individual proteins with specific functions in response to drought in *O. australiensis* indicate the importance of this species as a source of stress tolerance genes.

## 1. Introduction

More severe and sustained droughts are a consequence of accelerating climate change and therefore they represent a pressing threat to global food security, especially in non-irrigated crops. To ensure that the productivity of staple crops, such as rainfed rice, is maintained, there is a continual search for novel drought tolerance traits and their genetic basis. Drought stress results in a significant reduction in photosynthetic efficiency, growth, and crop yield. However, the extent of yield loss depends on many factors, among them the severity of drought stress and levels of crop tolerance.

As with several major crops, experimentation now extends beyond the familiar cultivated species; in the case of rice, this includes investigation into wild *Oryza* relatives [1]. The genus *Oryza* comprises 24 species, only two of which are widely cultivated, *O. sativa* (Asian rice) and *O. glaberrima* (African rice) [2]. *Oryza sativa* is widely cultivated from tropical to temperate zones, whereas *O. glaberrima* is traditionally grown in West Africa, although *O. sativa* cultivation is becoming steadily more dominant in Africa due to superior grain quality features, the ease of grain processing, and higher yield [3,4]. However, *O. glaberrima* has advantages over *O. sativa* in that it is generally more resistant to biotic and abiotic stresses, and therefore better adapted to respond to erratic climates [5]. It also has a considerable amount of wild *Oryza* genetic features through its progenitor, *O. barthii* [6]. There are several specific features of *O. glaberrima* that make it well adapted to drought stress, including a well-developed root system and reduced water loss through enhanced stomatal closure and leaf curling [4]. Therefore, it was a candidate for investigation of drought tolerance in the current study.

Undomesticated species of rice have more genetically heterogeneous populations compared with cultivated species. By necessity, cultivated rice has been subjected to continual selection and, through selfing, this has resulted in loss of many valuable genes. Numerous genes conferring resistance to biotic and abiotic stresses and/or boosting the yield of wild species have vanished in cultivated rice [7]. The penalty of the streamlining of gene expression for agricultural purposes is a loss of resilience in the stochastic natural environment, making wild rice species ideal candidates to enrich the gene pool of *O. sativa*. Genes that code for resistance to stressful environments, such as droughts, are of special interest in breeding upland rice suitable for adverse conditions. Numerous genes conferring resistance to biotic and abiotic stresses and/or boosting yield have been identified, mapped, and even transferred into high-yielding cultivars, such as the *Xa21* gene from *Oryza longistaminata,* which confers resistance to bacterial blight [8], and an *O. australiensis*-derived quantiative trait locus (QTL )conferring resistance to the brown planthopper (*Nilaparvata Lugens Stål*), one of the most destructive pests of Asian rice [9,10].

The *Oryza* genus is estimated to have diversified from a common ancestor approximately 15 million years ago [11]. *O. sativa* and *O. glaberrima* belong to the AA genome group whereas *O. australiensis* is the sole representative of the EE clade. The EE genome group is less closely related to the AA genome group than *O. sativa* and *O. glaberrima* are to each other [12]. Due to the low proportion of allelic diversity within species sharing the AA genome (including *O*. *sativa* [13]), previously unknown stress-related biomarkers that indicate drought tolerance are more likely to be found in wild relatives within more remote clades. The natural distribution of *O. australiensis* within the tropical savannah of northern Australia makes it a potentially valuable source of novel alleles and genes that confer tolerance to biotic and abiotic stresses [14]. Because *O*. *australiensis* is the unique species with the EE genome and is thus distantly related to species with the AA genome, it is the best candidate for the investigation of drought tolerance in rice.

Changes at the protein level in response to external stressors can provide a direct indicator of mechanisms of stress tolerance. In this context, quantitative proteomic analysis identifies abundance changes in stress-related protein biomarkers [15]. In a previous study we examined drought stress response in eight different varieties of *O. sativa* [16]. In this study, we performed a detailed quantitative proteomic analysis of *O. sativa* (cv. Nipponbare), *O. glaberrima*, and *O. australiensis* grown under control and drought stress conditions. *Oryza sativa* (cv. Nipponbare) is a domesticated cultivar with an available, well-annotated reference genome sequence, and is the most widely studied of all rice genotypes; it also has poor drought tolerance, having been selected from the northern Asian *japonica* sub-species. The wild species *O. australiensis* and the cultivated *O. glaberrima* (cv. CG14) contain rich untapped reservoirs of valuable genes and are therefore targeted for marker-assisted breeding [12,17]. The recent release of the *O. glaberrima* (cv. CG14) genome makes this the ideal candidate to represent this species [6]. Therefore, in this study, we performed a three-way comparison of proteomes from species that evolved in northern Asia, West Africa, and Oceania, with the aim of identifying both common and potentially novel drought-responsive protein accumulation signatures.

## 2. Results

### 2.1. Australian Rice Maintains Higher Leaf Water Potentials under Drought

**Figure 1:**
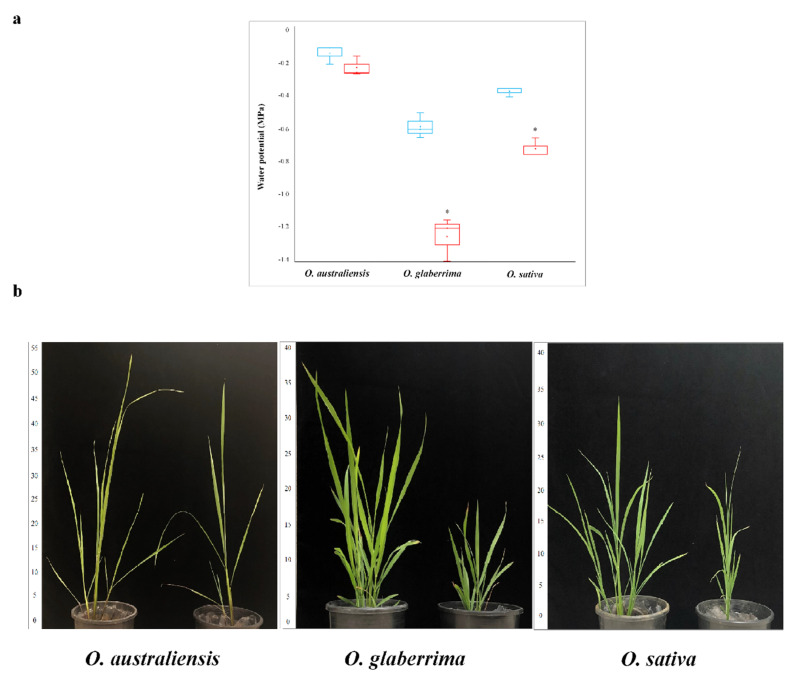
Leaf water potential of rice species under drought stress: (**a**) Leaf water potential (MPa) of youngest expanded leaves in control (blue) and drought stress (red) conditions. (**b**) Rice species used in this study grown under control (left) and drought stress (right) conditions. Asterisks indicate values which are statistically significantly different from controls, according to a Student’s *t*-test.

The measured water potentials of the youngest expanded leaf from well-watered and drought-stressed plants were statistically significantly different for both *O. sativa* and *O. glaberrima.* However, there was no impact of drought on water status in *O. australiensis*, where leaves maintained water potentials around −0.2 MPa even as the plants were growing in very water-restricted conditions (Figure 1a). The water potentials of *O. glaberrima* plants in drought were twice as large as those of *O. sativa* plants under drought stress, reflecting hydraulic differences between these species under drought which may be attributable to differences in stomatal behavior (Figure 1a).

The shoot and root morphology, as presented in Table 1 and Figure 1b, showed that the *O. australiensis* seedlings, while similar in mass to the other two species, were 65–70% taller. The shoot fresh mass of all three species was clearly reduced by drought stress, while the shoot dried mass was also reduced, but to a lesser extent. The root dried mass of *O. australiensis* was less than half of the other two species when grown under control conditions, and was not changed by drought stress. The root dried mass of *O. glaberrima* was also unchanged in drought conditions, while the root dried mass of *O. sativa* was reduced by 50% in the presence of drought stress. In contrast to the leaf water potential data, none of the observed morphological differences, in terms of shoot and root mass or shoot height, were statistically different between species or conditions, due to the variability between biological replicate plants.

### 2.2. Initial Proteome Characterization of Leaf Tissue from Plants Grown under Control and Drought Stress Conditions

We performed a label-free quantitative shotgun proteomic analysis that involved the analysis of leaf tissue from three biological replicates of each species of rice under control and drought stress conditions. Table 2 shows the number of proteins and peptides identified in each replicate analysis, as well as the number of proteins reproducibly identified in all three replicates, for each of the three species grown under control and drought-stress conditions. Further details on all proteins identified are provided in Supplementary Table S1. The number of reproducibly identified proteins, defined as those found to be present in all three replicates, varied from 934 to 1354 proteins. Combining the proteins identified in all six sample types, a non-redundant total of 1890 proteins was reproducibly identified from the leaf tissue of the three species. Comparing ion intensities between LC–MS analyses of all 18 samples enabled us to achieve the label-free quantitation of proteins between species. Details of all proteins reproducibly identified in this study are provided in Supplementary Table S1.

**Figure 2:**
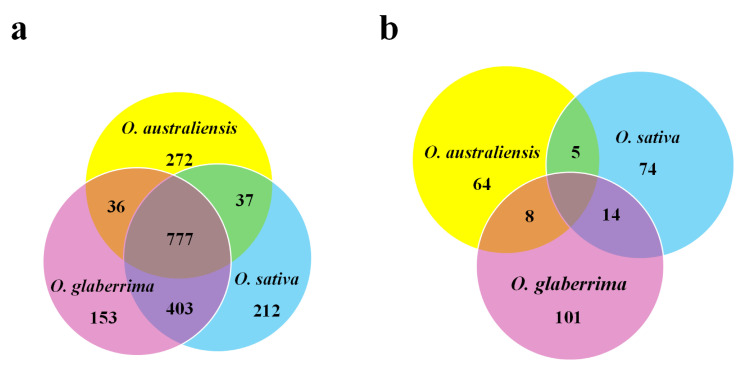
Profiling of identified proteins in three species of rice: (**a**) Venn diagram of reproducibly identified proteins from each species. (**b**) Venn diagram of proteins differentially changed in abundance in stress conditions from each species.

**Figure 3:**
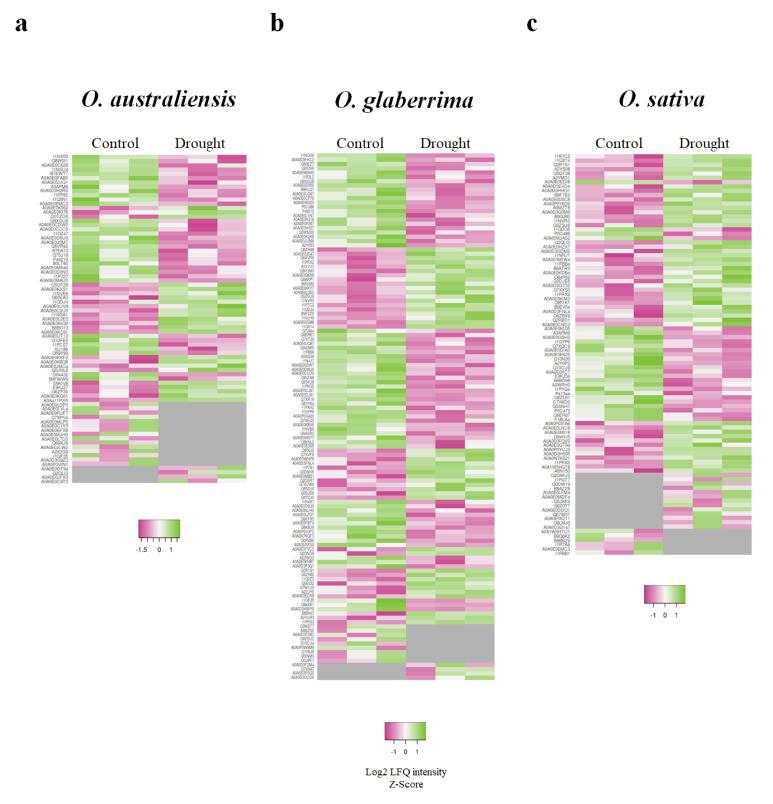
Heatmaps showing relative abundance intensity of proteins induced in response to drought stress across three species of rice. (**a**) *O. australiensis*, (**b**) *O. glaberrima*, and (**c**) *O. sativa*. The gray boxes show zero intensity. Columns indicate individual replicates.

The highest number of total proteins was identified in *O. sativa*, which also contained the lowest percentage of proteins with significantly changed levels of accumulation in drought conditions (Table 3). Although 777 proteins were common to all three species (Figure 2a), none was identified as significantly changed in protein accumulation in response to drought stress (Figure 2b). *O. australiensis* and *O. glaberrima* followed a similar trend, which contrasted with *O. sativa,* in that substantially more proteins decreased in abundance in response to drought than increased in abundance. As shown in Table 3 and Figure 3, there were 64, 101, and 74 proteins showing different levels of accumulation in response to drought stress in *O. australiensis, O. glaberrima*, and *O. sativa*, respectively. Notably, >90% of proteins identified showed no change in levels of protein accumulation in response to drought stress in all three species.

### 2.3. Proteins Exclusively Identified in Droughted and Well-Watered Leaf Tissues

**Figure 4:**
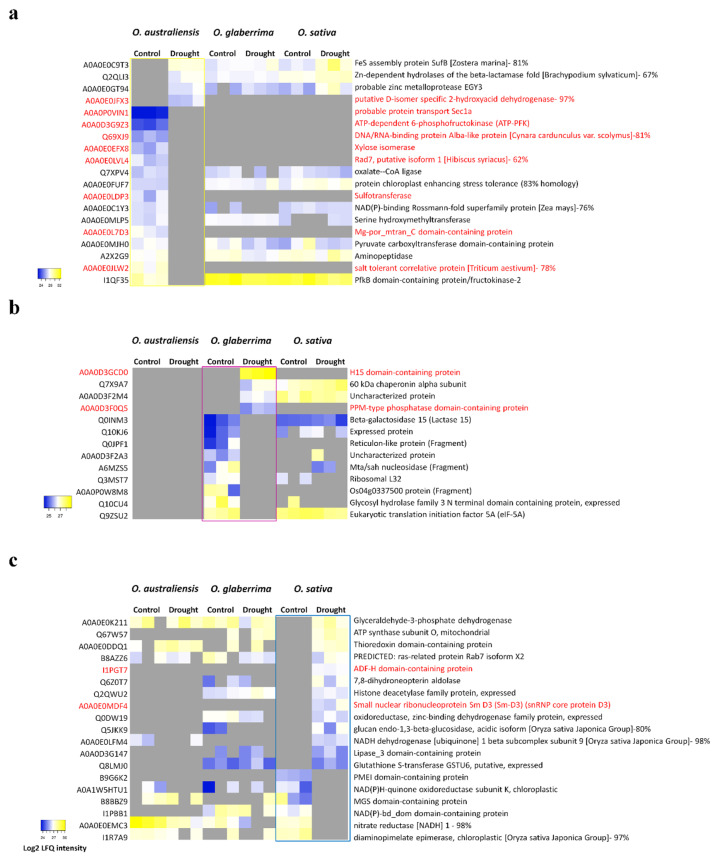
Heatmaps showing relative levels of accumulation of proteins exclusively identified under control and drought stress conditions in (**a**) *O. australiensis*, (**b**) *O. glaberrima*, and (**c**) *O. sativa*. The relative protein accumulation profiles in the other species are also shown. Protein names highlighted in red indicate proteins found uniquely in the indicated species.

Label-free quantitative shotgun proteomics revealed that a number of proteins were identified exclusively either in drought stress or well-watered control conditions. Figure 4 shows the profile of relative protein accumulation for those proteins found exclusively in each of the genotypes in each condition, along with their relative protein accumulation profile in all three species. Only four proteins were identified in stress conditions, but absent in control conditions, in *O. australiensis* and *O. glaberrima*. In contrast, 13 proteins were identified exclusively in drought stress, but not found in control conditions, in *O. sativa*. A greater number of proteins were present in unstressed leaf samples, but absent in droughted leaves, in both *O. australiensis* and *O. glaberrima* (15 and nine proteins, respectively) when compared with *O. sativa,* where only six proteins were found exclusively in well-watered samples. Interestingly, eight of the 15 such proteins in *O. australiensis* were uniquely identified in this species, and not in the other two species.

### 2.4. Gene Ontology Functional Classification of Significantly Altered Proteins in Response to Drought Stress

**Figure 5:**
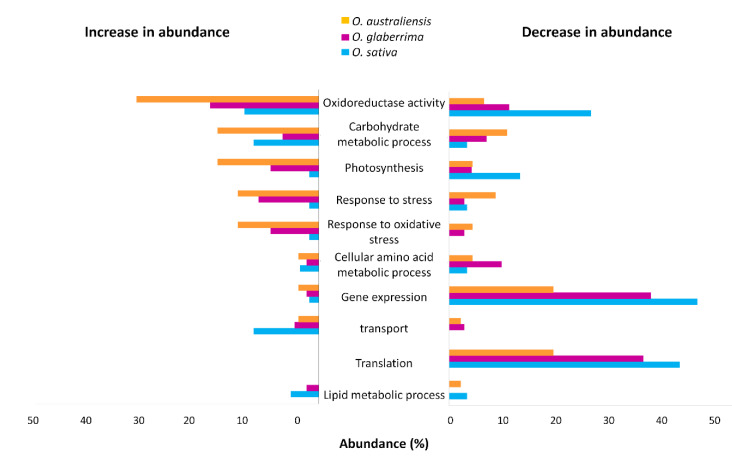
Functional classification of differentially accumulated proteins under drought stress conditions. The bars illustrate the percentage of proteins in 10 functional categories that are significantly changed under drought stress. Different colors represent different species of rice, as indicated.

Gene ontology functional classification was performed to explore the molecular function of all proteins significantly altered in expression by drought stress (Figure 5). Proteins that responded to stress were distributed across several functional categories, the most prominent of which were oxidoreductase activity, photosynthesis, response to stress, response to oxidative stress, and carbohydrate metabolic processes. One of the most apparent contrasts between *O. sativa* and the other two species in gene ontology functional classification was the relative increase in abundance of photosynthesis-associated proteins in both *O*. *australiensis* and *O*. *glaberrima* following drought treatment, whereas photosynthesis-associated proteins showed reduced levels of protein accumulation in *O. sativa*. Another obvious difference was the smaller number of stress-responsive proteins in *O. sativa* compared with *O*. *australiensis* and *O*. *glaberrima.* For example, only one protein in the response to stress category increased in abundance in stress conditions in *O*. *sativa* (probable glutathione S-transferase DHAR1) which was also significantly increased in abundance in *O*. *glaberrima*. A functionally related protein, L-ascorbate peroxidase 4, APX4 (I1QDJ4), significantly increased in abundance in both *O. glaberrima* and *O. australiensis.* It was identified as one of the most responsive proteins to drought stress, and the observed change in protein accumulation was very similar in both species.

Notably, all differentially accumulated proteins within the gene ontology functional category of protein translation decreased significantly in the stress condition in the three species, while none increased in abundance. Many of these are categorized as structural constituents of ribosomes, suggesting that a significant decrease in overall protein synthesis occurred in response to drought stress.

Seven proteins with increased abundance in response to stress in *O. sativa* were also predicted to be involved in molecular transport. Three of these were members of the non-specific lipid transfer protein (nsLTP) family. Another member of the nsLTP protein family was also identified in *O. glaberrima*. Ferritin, another protein in this category, was found in both *O. glaberrima* and *O. sativa*. In contrast, probable protein transporter Sec1a, which is also involved in membrane trafficking, was exclusively found in control conditions in *O. australiensis*, as shown in Figure 4.

Considering the proteins in the carbohydrate metabolism functional category, those affected by drought stress only in *O. australiensis* included ADP-glucose pyrophosphorylase (glucose-1-phosphate adenylyltransferase) and starch synthase.

### 2.5. GO Functional Classification of Significantly Altered Proteins in Response to Drought Stress

A total of 29 proteins with significantly altered accumulation levels in response to drought were identified only in one species, but not detected in either of the other two species. This included 18 proteins unique to *O. australiensis*, seven proteins unique to *O. sativa*, and four proteins unique to *O. glaberrima*. Many of these were proteins initially annotated with uncharacterized functions, but Blast searching and functional domain analysis enabled us to predict the function of most of the uncharacterized proteins based on sequence homology. Table 4 presents all 29 of these proteins, and includes the homologous proteins and functional domains identified for those proteins which did not have a clearly annotated initial function. Two uncharacterized proteins of *O. australiensis* were found to have a noticeably lower homology to known proteins, which suggests they may have potentially novel functions [18]. A0A0E0LIV9 was 44% identical to a thylakoid soluble phosphoprotein from *Carex littledalei* (an Asian sedge), and A0A0E0LVL4 was 56% identical to a ferredoxin-like protein from *Striga asiatica* (witchweed).

### 2.6. Parallel Reaction Monitoring (PRM) Validation

To verify the differential changes in abundance of drought stress-responsive proteins measured in our shotgun proteomics experiments, parallel reaction monitoring (PRM) analysis was performed on six proteins that showed differential abundance in response to drought stress. The proteins were selected to include two uncharacterized proteins found uniquely in *O. australiensis* (as mentioned in Section 2.5), one unique protein in *O. sativa* (ADF-H domain-containing protein), and three proteins which were significantly changed in at least two species (ferritin, 30S ribosomal protein S6 alpha, and pyruvate phosphate dikinase). These validation experiments were performed on additional aliquots of the same extracted peptide samples used for the discovery phase study.

**Figure 6:**
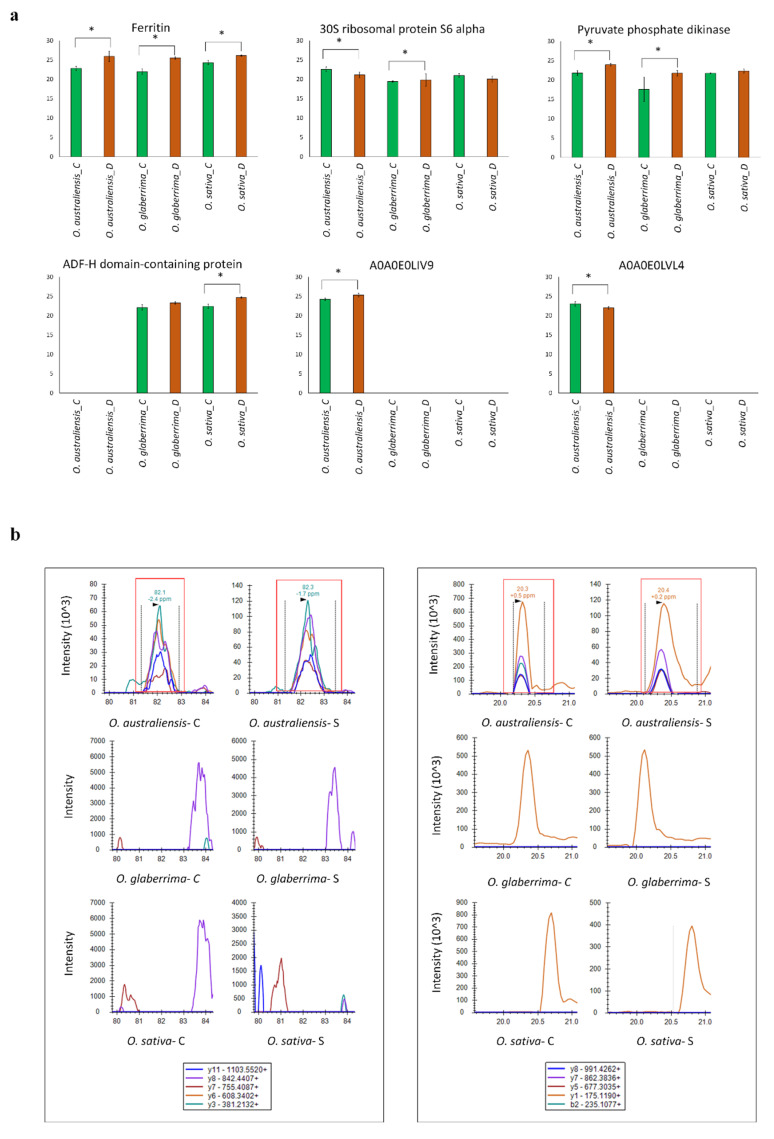
Parallel Reaction Monitoring (PRM) validation of drought stress-responsive proteins. (**a**) Bars illustrate the relative abundance of proteins (Log 2 area) in three rice species under control (C) and drought stress (D) conditions. An asterisk (*) indicates a statistically significant difference between the control and drought conditions, according to a Mann–Whitney U-test (*p*-values < 0.05). (**b**) PRM transitions used for targeted verification of AAGDGDGGFSFGGLFSK.

The results obtained by PRM were mostly in agreement with label-free shotgun proteomics, showing a similar change in abundance of the selected proteins in response to stress conditions. In agreement with the shotgun proteomics, uncharacterized proteins A0A0E0LIV9 and A0A0E0LVL4 were both successfully identified in *O. australiensis,* and not detected in the other two species (Figure 6). The targeted proteomics approach was successful in detecting peptides from some proteins which were reported as absent in the shotgun data, highlighting the greater sensitivity of analyte detection, which is expected when using a targeted approach [19]. Such a result was observed for the ADF-H domain-containing protein (I1PGT7), which was found uniquely in *O. sativa* in the label-free shotgun proteomics data, while PRM revealed the presence of one of the peptides of this protein (ALLTELQALEEHLK) in *O. glaberrima* (Figure 6). Similarly, although no peptides for A0A0E0LVL4 were identified in any of three replicates of droughted leaves of *O. australiensis* in the shotgun data, a targeted peptide (AYEGQCDQVR) was successfully identified in the stressed samples, albeit in lower amounts.

## 3. Discussion

### 3.1. Is Oryza australiensis a Source of Unique Drought-Stress Responsive Markers?

This study represents the first comparative proteomics analysis of rice species which extends beyond the two cultivated species into an important wild rice relative. Water potentials revealed that *O. australiensis* has a degree of hydraulic drought tolerance not seen in the other species. The tight control of leaf water status by the wild rice during drought may lessen the impact of drought upon the proteome of the plant cells, relative to the two cultivated species. It is also possible that differences in root architecture between the species may greatly influence their respective drought stress responses, as has been shown previously [20]. Such differences cannot be readily assessed in a study of the leaf proteome from plants grown in pots in greenhouse conditions, although there is some evidence that changes in root conditions can cause significant changes in the leaf proteome of rice [21].

On further investigation of proteins identified only in drought-stress conditions in *O. australiensis*, we observed that three of the four such proteins are predicted to be zinc metalloproteases: EGY3, probable zinc metalloprotease; Q2QLI3, which has 67% identity with Zn-dependent hydrolases of the beta-lactamase fold in *Brachypodium sylvaticum*; and SufB, an FeS cluster assembly protein. The first two of these also increased in droughted leaves in *O. sativa*, but not in *O. glaberrima*.

As the genome of *O. australiensis* has not yet been fully sequenced, it is possible that peptide to spectrum matching in this study may have missed some important peptides and proteins. If there are insufficient sequence data available for one species when performing a cross species comparison, the number of peptide to spectrum matches is lower, because the software cannot find a good match for a significant number of good quality spectra [22]. However, the concordance across the three species in terms of the number of peptide to spectrum matches performed and the number of peptides identified, as shown in Table 2, suggests this is not a significant issue in our results.

In order to compensate for the incomplete genome sequence of this species, peptide to spectrum matching was performed against sequences from all available species of the genus *Oryza*, as described in the Materials and Methods. From an evolutionary point of view, our results indicate that *O. australiensis*, which has an EE genome, is closer to *O. punctata*, which has a BB genome, as has been reported previously [23,24]. Many MS/MS spectra in *O. australiensis* were matched exclusively with sequences from *O. punctata*. Out of 18 uniquely changed proteins in response to stress which represent potential biomarkers for drought tolerance (Table 4), eight were exclusively annotated from sequences found in *O. punctata*.

### 3.2. Photosynthetic Function Appears to Be Resilient under Drought in O. australiensis

A major proportion of the leaf protein complement is associated with photosynthesis, accounting for 17.6% of the proteins with known ontology which increased in abundance in drought stress (Figure 5). Gene ontology analysis showed that five proteins that increased in abundance in response to stress in *O. australiensis* were categorized as carbohydrate metabolism proteins (Section 2.4). Two of these are known from previous studies to be involved in starch biosynthetic processes: ADP-glucose pyrophosphorylase (AGP) and starch synthase (D0TZD6). Both are key regulatory enzymes in starch biosynthesis, with AGP catalyzing the first step in starch biosynthesis [17]. The allosteric regulation of AGP by 3-phosphoglycerate and inorganic phosphate makes this enzyme a key regulator of starch biosynthesis.

The increased accumulation of proteins related to carbohydrate biosynthesis was observed in drought stress in *O. sativa*, but none of them was involved in starch biosynthesis. Hence, we can postulate that the increased abundance of starch biosynthesis and photosynthesis-associated proteins appears to be correlated with increased tolerance to drought in *O. australiensis*. This correlation applies only at the protein accumulation level, and further experimental studies are required in order to determine whether a similar correlation exists at a functional level.

### 3.3. Stress-Response Proteins Are Relatively More Abundant in O. glaberrima and O. australiensis than in O. sativa

Once water deficits perturb cellular homeostasis, adjustment is required to ensure the recovery of plants after stress exposure. The adjustment of cell homeostasis through changes in the steady state level of proteins, such as by reactive oxygen species (ROS)-scavenging enzymes, is one essential strategy of plants to tolerate stress [25]. In this study, with exposure to drought stress, the greatest number of induced stress-response proteins was in *O. glaberrima,* which also had the largest depression of leaf water status. Different isoforms of L-ascorbate peroxidase (APX), including I1QDJ4 and A0A0E0GKX8, increased in droughted leaves in *O. glaberrima*. In *O. australiensis,* two isoforms of APX also increased in abundance in response to drought stress, I1QDJ4 and A0A0E0KC91. It has been demonstrated previously that APX4 in *O. sativa* plays a role in leaf senescence processes mediated by ROS signaling, in addition to its well-known function as an antioxidant in peroxide scavenging [26], and is also directly involved in the protection of plant cells against adverse environmental conditions [27]. The remaining stress-response proteins in *O. glaberrima* have also been shown to be involved in the enhanced tolerance of plants to stress conditions, including a protein with 98% homology to a probable glutathione S-transferase with dehydroascorbate reductase activity in *O. sativa* (DHAR1), osmotin-like protein, and a protein with 85% homology to 3-beta hydroxysteroid dehydrogenase from *Zea mays*. Previous studies have shown that the overexpression of a *Liriodendron chinense* DHAR gene in *Arabidopsis* led to increased abscisic acid levels, as well as enhanced tolerance to salt and drought stress [28], and DHAR1 and DHAR3 in *Arabidopsis* were differentially expressed in response to various stresses, including high light, salt, and cold stresses [29]. The overexpression of a similar osmotin-like protein has been shown to confer tolerance against abiotic stresses in sesame [30], while the overexpression of a 3-beta hydroxysteroid dehydrogenase in *Arabidopsis* was found to increase growth rate and tolerance to salt stress [31]. This indicates that *O. glaberrima* should not be overlooked as a source of drought-response biomarkers; in spite of the selective processes it has undergone, there are clearly stress-tolerance traits to be identified.

### 3.4. Ribosomal Proteins Show Reduced Accumulation in Response to Drought Stress

Ribosomal proteins (RPs) are an integral protein part of ribosomes, which are known not only for playing crucial roles in ribosome assembly and protein synthesis, but also in various other different developmental processes [32,33,34]. It has been shown that RPs are differentially regulated in response to environmental conditions, and the transcriptional repression of genes responsible for ribosomal protein biosynthesis has been reported in plant cells during stress conditions [35]. Our results showed that all significantly changed proteins in *O. sativa* and *O. australiensis* categorized as translation-related proteins were structural constituents of ribosomes. In *O. glaberrima*, the majority of such proteins were categorized as structural constituents of ribosomes, along with three other proteins: eukaryotic translation initiation factor 5A (eIF-5A), tyrosyl-tRNA synthetase, and aminoacyl-tRNA synthase. It appears that ribosomal protein synthesis may be suppressed at the transcriptional level in all three species in response to drought stress conditions, manifesting as a decreased abundance of ribosomal proteins. The decrease in abundance of RPs in response to drought stress correlates with the fact that ribosome biogenesis is an energy-demanding process [36,37,38]. On the other hand, it has also been reported that RPs might be partially responsible for stress tolerance in plants. Previous studies have shown that the overexpression of a 23-kDa ribosomal protein in rice plants was linked to enhanced water use efficiency and increased tolerance to drought stress [39], while the decreased expression of a 32-kDa ribosomal protein was observed in salt-stressed rice seedlings [40]. Similar findings have been reported in *Arabidopsis*, where plastid ribosomal proteins were increased after exposure to short-term salt stress [41], and also in wheat, where expression levels of ribosomal proteins were found to be different between drought-tolerant and drought-susceptible genotypes [42,43]. The exact nature of the correlation between the abundance of RPs and the drought tolerance of a rice species is still not clear and further investigation is warranted.

## 4. Materials and Methods

### 4.1. Plant Material and Sample Preparation

Three different species of rice—*Oryza sativa* (cv. Nipponbare), *Oryza australiensis*, and *Oryza glaberrima*—were sown in individual small pots containing 1 kg of Robertson soil containing NPK 23:3.95:14 fertilizer. The experiment was performed at the Plant Growth Facility of Macquarie University (North Ryde, NSW, Australia) with a 12 h/12 h light/dark photoperiod under a light intensity of 700 μmol m^−2^ s^−1^ at 28/22 °C day/night. Plants were well watered for 30 days, followed by applying drought stress until the field capacity (FC) reached 40%. The rate of evapotranspiration was monitored on a daily basis by weighing the pots, and the amount of water lost was replaced, up to 40% FC. The stressed plants were maintained at 40% FC for 7 days. Leaves were collected from treated and control plants and immediately frozen in liquid nitrogen, followed by storage at −80 °C prior to subsequent analysis.

### 4.2. Leaf Water Potential (LWP) Measurements

LWP was measured at midday on the youngest fully expanded leaves using a pressure chamber (PMS Instrument Company, Albany, OR, USA). The leaf was enclosed in aluminum foil and cut around the petiole close to the leaf base. The cut leaf was immediately placed in the pressure chamber, followed by pressurizing the chamber. The water potential was then recorded after the bubbling of water was observed in the phloem vessels.

### 4.3. Protein Extraction and Protein Assay

Fifty mgf leaf tissue was ground to a powder in liquid nitrogen, and this was repeated for leaf tissue collected from each of three biological replicate plants. Proteins were extracted using trichloroacetic acid–acetone extraction. Briefly, leaf powder was suspended in 1.5 mL of extraction medium (10% trichloroacetic acid in acetone, 2% β-mercaptoethanol), vortexed for 30 min at 4 °C, and incubated at −20 °C for 45 min. The pellet was collected after centrifugation at 16,000× *g* for 30 min, and washed three times with 100% ice-cold acetone, followed by centrifugation at 16,000× *g* for 30 min. The pellet was lyophilized in a vacuum centrifuge and resuspended in 3% SDS in 50 mM Tris-HCl (pH8.8). Samples were then precipitated by methanol–chloroform. The pellet was suspended in 8 M urea in 100 mM Tris-HCl, pH 8.8, and the protein concentration was measured by bicinchoninic acid assay (Thermo Fisher Scientific, San Jose, CA, USA).

### 4.4. In-Solution Digestion and Peptide Extraction

Proteins were diluted five-fold using 100 mM Tris-HCl (pH 8.8), then reduced and alkylated with 10 mM dithiothreitol at room temperature for 1 h, followed by 20 mM iodoacetamide at room temperature in the dark for 45 min. Proteins were digested using trypsin at 37 °C overnight. The enzyme was deactivated by the addition of formic acid to 1% of the total volume, followed by desalting the peptides using a stage tip packed in-house with Empore SDB-RPS (Sigma-Aldrich, St. Louis, MO, USA). Samples were eluted from stage tips using 200 μL of 80% acetonitrile/5% ammonium hydroxide. The peptides were vacuum dried, resolubilized in 0.1% formic acid, and quantified by micro-BCA assay (Pierce Biotechnology, Rockford, IL, USA).

### 4.5. Nano LC–MS/MS

Peptides were analyzed by nanoflow LC–MS/MS using a Q Exactive Orbitrap mass spectrometer (Thermo Fisher Scientific, San Jose, CA, USA) coupled to an EASY-nLC1000 nano-flow HPLC system (Thermo Fisher Scientific, San Jose, CA, USA). Reversed phase columns of 75 µm internal diameter were packed in-house to 10 cm length with ES-C18 Halo^®^, 2.7 µm, 160 Å, (Advanced Materials Technology, Wilmington, DE, USA). Peptides were eluted from the column for 130 min, starting with 100% buffer A (0.1% formic acid), using a linear solvent gradient, with steps from 2 to 30% of buffer B (99.9% (*v/v*) ACN, 0.1% (*v/v*) formic acid) for 120 min and 30 to 85% of buffer B for 10 min. One full MS scan over the scan range of 350 to 1850 m/z was acquired in the Orbitrap at a resolution of 70,000 after accumulation to an automated gain control (AGC) target value of 1 × 10^7^. MS/MS analysis was conducted for the 10 most intense ions. The maximum injection time was set to 60 ms and higher-energy collisional dissociation fragmentation was performed at 27% normalized collision energy, with selected ions dynamically excluded for 20 s.

### 4.6. Parallel Reaction Monitoring (PRM) Analysis

Parallel reaction monitoring (PRM) analysis was carried out using the same tandem mass spectrometry system for the analysis of six differentially accumulated proteins. Skyline software version 20.1.0.76 was used for PRM analysis [44]. An inclusion list containing the mass to charge ratio of the unique precursor peptides of interest according to the results of our quantitative proteomic analysis was generated and used for PRM analysis. The inclusion list started targeted scans at a resolving power of 17,500, an AGC target of 2 × 10^5^, a maximum injection time of 100 ms, and a normalized collision energy of 30% in HCD. All peaks were manually inspected to ensure that the correct ions were selected. The proteins were quantified using the peak areas for the transitions in the respective peptides and proteins. Fragment ion peak areas were normalized with one of the most abundant peptides belonging to ribulose bisphosphate carboxylase large chain (RuBisCO). Peak areas are reported as the average of three replicate experiments, with error bars showing the standard deviation. Prior to statistical analysis, the normalized peak areas were log2 transformed. Differentially expressed proteins were identified using the Mann–Whitney U-test to compare the stress versus control condition for each of the species.

### 4.7. Protein Identification and MS Data Analysis

Raw MS data files were analyzed using MaxQuant 1.6.5.0 for peptide to spectrum matching and label-free quantitation (LFQ) [45]. FASTA files of protein sequences from 15 species of *Oryza* genus containing a total of 297,554 proteins were downloaded from UniProt in June 2019 and assembled into a single database for peptide to spectrum matching. This included *O. sativa*, *O. glaberrima, O. australiensis*, *O. meridionalis*, *O. punctata*, *O. nivara*, *O. barthii*, *O. rufipogon*, *O. meyeriana*, *O. officinalis*, *O. coarctata*, *O. longistaminata*, *O. glumaepatula*, *O. ridleyi*, and *O. schlechteri*. Fixed modification with cysteine carbamidomethylation was used, and the variable modifications considered included methionine oxidation, protein N-terminal acetylation, asparagine and glutamine deamination, and N-terminal glutamine to pyroglutamate conversion. The false discovery rate was set at 1% for proteins and peptides and estimated using a reversed sequence database. Trypsin enzymatic specificity was applied, allowing a maximum of two missed cleavages [45,46]. The precursor and fragment mass tolerances were set to 0.5 Da and 20 ppm, respectively. Matching between runs was performed with a match time window of 0.7 min and a retention time alignment window of 20 min.

### 4.8. Statistical Analysis

Statistical analysis and data processing of MaxQuant was performed in Perseus 1.6.0.2 [47]. Total identified proteins in each species were filtered based on those that were quantified in all three replicates of either the control or drought stress condition. These are annotated as reproducibly identified proteins in Table 2. Label-free quantitation (LFQ) intensities were log transformed and missing values were considered as 0 for mathematical purposes. A pairwise comparison between control and stress conditions was performed via two-tailed *t*-tests. Differentially accumulated proteins were identified considering a threshold of >1.5- or <0.6-fold change and a *p*-value < 0.05. Fold changes were expressed as a ratio of the quantitative value of proteins present under drought stress with that of the control, on a linear scale. Heat maps were generated using the log2 LFQ intensity. Hierarchical clustering was performed using Euclidean as the distance metric and the average as the linkage criterion. All mass spectrometric proteomic data have been deposited into the PRIDE data repository [48] and are available via ProteomeXchange with project accession number PXD019885.

### 4.9. Functional Protein Annotation

Gene ontology (GO) annotation was acquired from the UniProt database, matched to the list of differentially expressed proteins and classified into functional categories using PloGO [49]. GO annotation was summarized for each category of interest from a list of selected GO categories, for the results of the pairwise comparison, including proteins differentially increased or decreased in abundance. InterProScan 5, incorporating Pfam and other tools, was used for functional domain analysis [50].

## 5. Conclusions

The proteomic and physiological responses of three species of rice were investigated using label-free quantitative shotgun proteomics and PRM after the exposure of plants to drought stress. *Oryza australiensis* was clearly different from the other two species in terms of leaf water potential, which was barely changed in response to drought stress, and in terms of the overall protein accumulation signature. At the protein accumulation level, the only molecular process that appeared to be equally affected in all three species was translation, which was globally suppressed, suggesting a generalized reduction in protein synthesis occurring in response to drought stress. Photosynthetic efficiency was not affected by stress in *O. australiensis*, whereas it was distinctly impacted in *O. sativa*. Moreover, none of the differentially accumulated proteins was significantly altered in all three species simultaneously, although some were found to be changed at the protein accumulation level in two out of the three species. This suggests that phylogenetic distance between species may be reflected in the differences observed between species at the proteome level. It appears that *O. australiensis* may contain the largest repertoire of novel stress responsive genes, as the overall protein accumulation profile in this species was quite distinct from that of the other two species examined. This included a significantly altered accumulation of proteins in response to drought stress that are essentially uncharacterized and have no annotated function; these warrant further detailed investigation. The accumulation pattern of two of the most interesting uncharacterized proteins, in terms of having minimal sequence homology with other plants, were validated by PRM data. Taken together, the proteomics information generated in this study has the potential to be used in selective breeding in *O. sativa*, thus contributing to improvements in crop yield in cultivated rice.

## Figures and Tables

**Table 1:** Summary of seedling height and mass data collected for three species of rice.

Species and Status	Shoot Fresh Mass, Average ± SD (g)	Shoot Dried Mass, Average ± SD (g)	Root Dried Mass, Average ± SD (g)	Shoot Height, Average ± SD (cm)
*O. australiensis - C*	3.91 ± 0.43	0.57 ± 0.09	0.21 ± 0.06	48.67 ± 3.51
*O. australiensis - D*	1.62 ± 0.85	0.35 ± 0.11	0.23 ± 0.06	42.33 ± 11.93
*O. glaberrima - C*	3.60 ± 1.85	0.68 ± 0.31	0.52 ± 0.06	28.00 ± 4.00
*O. glaberrima - D*	2.38 ± 0.56	0.65 ± 0.14	0.57 ± 0.13	24.67 ± 4.04
*O. sativa - C*	3.19 ± 1.50	0.68 ± 0.34	0.60 ± 0.33	29.67 ± 4.04
*O. sativa - D*	1.87 ± 0.25	0.55 ± 0.05	0.30 ± 0.06	27.33 ± 2.52

C = control conditions, D = drought conditions.

**Table 2:** Summary of protein identification data of leaf samples for three species of rice.

Row	Rice Species- Treatment	Proteins			Peptides			Protein RSD ^b^ (%)	Peptide RSD (%)	Reproducibly Identified Proteins
		R1 ^a^	R2	R3	R1	R2	R3			
1	*O. australiensis*- C ^c^	1411	1324	1219	9341	8755	7821	7.3	8.9	1033
2	*O. australiensis*- D	1299	1128	1272	8667	7625	8580	7.5	7.0	934
3	*O. glaberrima*- C	1574	1513	1656	8860	8925	9342	4.5	2.9	1230
4	*O. glaberrima*- D	1522	1519	1626	8985	8829	9465	3.9	3.6	1213
5	*O. sativa*- C	1545	1463	1539	8285	8126	8435	3.2	1.9	1160
6	*O. sativa*- D	1541	1698	1697	8197	9698	9571	5.5	9.1	1354

^a^ R1,2,3 refer to biological replicates. ^b^ RSD, relative standard deviation. ^c^ C refers to control conditions, D refers to drought conditions.

**Table 3:** Summary of numbers of proteins showing different levels of protein accumulation between drought stress and control conditions in three species of rice.

Row	Rice Species	Total	Unchanged	Increased	Decreased	%Changed
1	*O. australiensis*	1122	1045	28	49	6.8
2	*O. glaberrima*	1369	1246	47	76	9.0
3	*O. sativa*	1429	1336	61	32	6.5

**Table 4:** Proteins with significantly altered accumulation levels in response to drought stress which were uniquely identified in one species.

Species	Row	UniProt ID	UniProt Protein Name	Phytozome Gene Name	Fold Change	Homologous Protein	Identity (%)	Functional Domain(s)
	1	A0A0E0JFX3	Uncharacterized protein	LOC_Os01g12830.1 erythronate-4-phosphate dehydrogenase domain-containing protein	26.7	Putative D-isomer specific 2-hydroxyacid dehydrogenase (*Oryza sativa*)	97%	D-isomer specific 2-hydroxyacid dehydrogenase (2HADH), NAD-binding domain; 2HADH, catalytic domain
	2	D0TZD6	Starch synthase	LOC_Os07g22930.2 starch synthase	1.9	- ^a^	-	Glycosyl transferase group 1; Starch synthase catalytic domain
	3	A0A0E0LIV9	Uncharacterized protein	LOC_Os07g13969.1 expressed protein	1.7	Thylakoid soluble phosphoprotein TSP9 (*Carex littledalei*)	44%	Thylakoid soluble phosphoprotein TSP9
***O. australiensis***	4	A0A0E0KBQ8	FBPase domain-containing protein	LOC_Os03g16050.1 fructose-1,6-bisphosphatase	1.3	Fructose-1,6-bisphosphatase	97%	Fructose-1-6-bisphosphatase (FBPase), N-terminal domain; FBPase C-terminal domain
	5	A0A0E0KXS1	Uncharacterized protein	LOC_Os05g02530.1 glutathione S-transferase, N-terminal domain-containing protein	1.0	Probable glutathione S-transferase DHAR1 (*Oryza sativa*)	98%	Glutathione S-transferase (GST), N-terminal domain; GST, C-terminal domain
	6	A0A0E0JMC4	Peroxiredoxin	LOC_Os01g48420.1 peroxiredoxin	0.8	-	-	Redoxin
	7	A0A0E0KKF2	Uncharacterized protein	LOC_Os03g59100.1 pheophorbide a oxygenase, chloroplast precursor	0.7	Protochlorophyllide-dependent translocon component 52 (*Oryza sativa*)	88%	Pheophorbide a oxygenase; Rieske [2Fe-2S] domain
	8	A0A0E0C629	Peptidase A1 domain-containing protein	LOC_Os01g48740.1 aspartyl protease family protein	−0.7	Aspartic proteinase (*Oryza sativa*)	98%	Xylanase inhibitor N-terminal
	9	B0LT90	Triosephosphate isomerase	LOC_Os01g05490.1 triosephosphate isomerase, cytosolic	−0.7	-	-	Triosephosphate isomerase
	10	O04432	Glycine-rich protein	LOC_Os12g43600.1 RNA recognition motif-containing protein	−1.4	-	-	RNA recognition motif (RRM, RBD, or RNP domain)
	11	A0A0P0VIN1	Os02g0452500 protein	LOC_Os02g25580.1 Sec1 family transport protein	−22.7	Probable protein transport Sec1a (*Oryza sativa*)	98%	Sec1 family
	12	A0A0D3G9Z3	ATP-dependent 6-phospho-fructokinase	LOC_Os05g44922.1 6-phosphofructokinase	−24.1	-	-	Phosphofructokinase
	13	Q69XJ9	Os06g0602600 protein	LOC_Os06g40040.1 protein of unknown function domain-containing protein	−25.7	Alba domain-containing protein (*Cephalotus follicularis*)	78%	Alba
	14	A0A0E0EFX8	Xylose isomerase	LOC_Os07g47290.1 xylose isomerase	−26.3	-	-	Xylose isomerase-like
	15	A0A0E0LVL4	Uncharacterized protein	LOC_Os08g35710.1 expressed protein	−26.7	Ferredoxin-like protein (*Striga asiatica*)	56%	ND ^b^
	16	A0A0E0LDP3	Sulfotransferase	LOC_Os06g42120.1 sulfotransferase domain-containing protein	−26.8	-	-	Sulfotransferase domain
	17	A0A0E0L7D3	Mg-por_mtran_C domain-containing protein	LOC_Os06g04150.1 magnesium-protoporphyrin O-methyltransferase	−27.3	-	-	Magnesium-protoporphyrin IX methyltransferase C-terminus
	18	A0A0E0JLW2	Uncharacterized protein	LOC_Os01g46600.1 seed maturation protein PM41	−28.2	Salt-tolerant correlative protein (*Triticum aestivum*)	78%	ND
	19	A0A0D3GCD0	H15 domain-containing protein	LOC_Os06g04020.1 histone H1	28.6	-	-	Linker histone H1/H5
***O. glaberrima***	20	A0A0D3F0Q5	PPM-type phosphatase	LOC_Os02g05630.1 protein phosphatase 2C	25.7	-	-	Protein phosphatase 2C
	21	Q0JPF1	Reticulon-like protein B1	LOC_Os01g12650.1 reticulon domain-containing protein	−25.4	-	-	Reticulon
	22	A0A0P0W8M8	Probable aldo-keto reductase 1	LOC_Os04g26870.1 oxidoreductase, aldo/keto reductase family protein	−26.6	-	-	Aldo/keto reductase family
***O. sativa***	23	I1PGT7	ADF-H domain-containing protein	LOC_Os03g60580.1 actin-depolymerizing factor	26.7	-	-	Cofilin/tropomyosin-type actin-binding protein
	24	A0A0E0MDF4	Small nuclear ribonucleoprotein Sm D3 (Sm-D3)	LOC_Os02g01250.1 LSM domain-containing protein	26.5	-	-	LSM domain
	25	B8ATR3	Uncharacterized protein	LOC_Os04g34600.1 abscisic stress-ripening protein	1.9	Glycine-rich cell wall structural protein 2-like isoform X1 (*Panicum hallii*)	56%	ABA/WDS induced protein
	26	Q94HJ5	Putative 3-beta hydroxysteroid dehydrogenase/isomerase protein	LOC_Os05g01970.4 NAD-dependent epimerase/dehydratase family protein	0.8	-	-	NAD(P)H-binding
	27	Q7XQC9	Uncharacterized protein	LOC_Os04g02050.1 bifunctional 3-phosphoadenosine 5-phosphosulfate synthetase	−0.7	ATP sulfurylase 2-like (*Oryza brachyantha*)	96%	PUA-like domain; ATP-sulfurylase
	28	P41344	Ferredoxin--NADP reductase, leaf isozyme 1, chloroplastic	LOC_Os06g01850.1 ferredoxin--NADP reductase, chloroplast precursor	−0.9	-	-	Oxidoreductase NAD-binding domain; Oxidoreductase FAD-binding domain
	29	B9G6K2	PMEI domain-containing protein	LOC_Os10g36500.2 invertase/pectin methylesterase inhibitor family protein	−25.7	-	-	Plant invertase/pectin methylesterase inhibitor

^a^ (–) indicates a well characterized protein, with no homologous protein identity required; ^b^ none detected.

## Supplementary Materials

Supplementary materials can be found at https://www.mdpi.com/1422-0067/21/17/5980/s1. Table S1: Details of all proteins reproducibly identified in this study.

## Data Access

All data have also been submitted to the PRIDE data repository, and are available via ProteomeXchange with project identifier PXD019885.

## Abbreviations

FC	Field capacity
GO	Gene ontology
LFQ	Label-free quantitation
LWP	Leaf water potential
nLC–MS/MS	nanoflow liquid chromatography–tandem mass spectrometry
PRM	Parallel reaction monitoring
nsLTP	Non-specific lipid transfer protein
ROS	Reactive oxygen species
RP	Ribosomal protein
RSD	Relative standard deviation
